# The Interplay of Prolactin with Inflammatory Nutritional Markers and NT-proBNP in Chronic Kidney Disease

**DOI:** 10.3390/ijms26136347

**Published:** 2025-07-01

**Authors:** Crina Claudia Rusu, Diana Moldovan, Alina Potra, Dacian Tirinescu, Maria Ticala, Yuriy Maslyennikov, Andrada Barar, Alexandra Urs, Cosmina Ioana Bondor, Ana Valea, Ina Kacso

**Affiliations:** 1 Department of Nephrology, University of Medicine and Pharmacy “Iuliu Hatieganu” Cluj, 8 Victor Babes Street, 400012 Cluj-Napoca, Romania; 2 Department of Nephrology, County Emergency Clinical Hospital Cluj, 3-5 Clinicilor Street, 400006 Cluj-Napoca, Romania; 3 Department of Medical Informatics and Biostatistics, University of Medicine and Pharmacy “Iuliu Hatieganu” Cluj, 6 Pasteur Street, 400349 Cluj-Napoca, Romania; 4 Department of Endocrinology, University of Medicine and Pharmacy “Iuliu Hatieganu” Cluj, 8 Victor Babes Street, 400012 Cluj-Napoca, Romania; 5 Endocrinology Clinic, County Emergency Clinical Hospital Cluj, 400347 Cluj-Napoca, Romania

**Keywords:** prolactin, N-terminal pro-B-type natriuretic peptide, CKD, CVD, inflammation, biochemical markers

## Abstract

In chronic kidney disease (CKD), various disorders occur that worsen with the progression of CKD. These include increased levels of hormones such as adiponectin, leptin, and prolactin, changes in feedback loops and metabolism, and decreased renal clearance, contributing to significant morbidity and mortality. We conducted a cross-sectional observational study on 157 randomly selected patients with various stages of chronic kidney disease, 29% of whom had diabetes. We recorded clinical and usual laboratory data. We determined muscle mass and adipose tissue mass using bioimpedance. In addition, we measured serum prolactin levels, tumor necrosis factor-alpha (TNF-α), Interleukin 6 (IL-6), and Interleukin-1 beta (IL-1β). N-terminal pro-B-type natriuretic peptide (NT-proBNP) was evaluated as a marker of cardiac function. We evaluated the relation between prolactin, TNF-α, IL-6, IL-1β, and NT-proBNP by bivariate and multivariate analysis. In bivariate analysis, we recorded associations of prolactin with inflammatory markers: TNF-α (r = 0.65, *p* < 0.001), IL-6 (r = 0.66, *p* < 0.001), and IL-1β (r = 0.25, *p* = 0.002). In multivariate analysis we observed that serum prolactin values are associated with IL-1β [median (25th–75th percentile): [−0.001 (−0.001; −0.00003), *p* = 0.037], muscle mass [−0.03 (−0.04; −0.01), *p* = 0.003], and NT-proBNP [0.0001 (0.0001; 0.0001)] *p* < 0.001 In conclusion, in chronic kidney disease, prolactin is associated with inflammatory markers (IL-1β, TNF-α, IL-6), and nutritional status. Additionally, prolactin has been linked to NT-ProBNP, a marker of cardiac function.

## 1. Introduction

In chronic kidney disease, various disturbances in the body’s homeostasis occur, which worsen as the disease progresses and contribute to significant morbidity and increased mortality. Among these disturbances, accumulation of hormones such as adiponectin, leptin, and prolactin has been observed, due to changes in feedback loops, and by altering their metabolism and decreasing renal clearance [1].

Prolactin is a polypeptide hormone synthesized and secreted primarily by the lactotroph cells of the pituitary gland [2]. However, adipose tissue, the central nervous system, the immune system, the uterus, and the mammary glands can also produce prolactin. It has a role in lactation and other functions essential for maintaining homeostasis [3]. The effects of prolactin are due in part to the presence of its receptor in different tissues at different locations. Biological processes associated with hyperprolactinemia include insulin resistance [4,5], metabolic syndrome [6], and modulation of inflammation [7,8]. Additionally, prolactin has been shown to affect the cardiovascular and skeletal systems [9]. It is positively associated with cardiovascular mortality [10,11], induces myocardial injury, and is implicated in the pathogenesis of peripartum cardiomyopathy [12]. Prolactin also has numerous roles in the vascular system. Prolactin levels are positively correlated with blood pressure, are implicated in endothelial dysfunction, and contribute to the acceleration of atherosclerosis [6,7]. There are two significant prolactin isoforms: 23 kDa prolactin (also known as full-length prolactin) and the vasoinhibins, which include fragments ranging from 5.6 to 18 kDa [2,13]. Both act as circulating hormones and cytokines to stimulate or inhibit vascular formation and neovascularization [14], as well as endothelial cell proliferation and migration, protease production, and apoptosis [2].

In chronic kidney disease, serum prolactin levels increase as the estimated glomerular filtration rate (eGFR) decreases. Thus, hyperprolactinemia in these patients becomes highly prevalent, ranging from 30% in the early stages of CKD to 65% in those on hemodialysis (HD) [15]. Prolactin increases in the serum with loss of renal function through several mechanisms, including accumulation, reduced metabolism [16], and increased secretion by lactotrophs in the uremic state. In addition, in CKD, reduced dopamine availability in the brain directly stimulates prolactin secretion [17]. Carrero et al. observed that hyperprolactinemia and endocrine disorders in chronic kidney disease exhibit interesting associations with inflammation, endothelial dysfunction, arterial stiffness, protein–energy wasting, and other cardiometabolic disorders that contribute to the excess mortality associated with CKD [18]. Other authors have also considered prolactin to be a uremic toxin with cardiovascular effects in CKD [15,19]. However, further clarification is needed regarding the role and place of prolactin in cardiovascular disease in chronic kidney disease. It is not yet conclusively established whether hyperprolactinemia is a cardiovascular risk factor or an intermediary in a major pathophysiological pathway [19,20]. A recent study found that prolactin levels may not substantially influence the relationship between chronic kidney disease and cardiovascular disease [20]. Furthermore, lifestyle, in conjunction with regional dietary habits and microbiota characteristics, may influence prolactin levels and their interactions in chronic kidney disease across different population groups.

Therefore, this study aims to evaluate the factors associated with prolactin levels in individuals with chronic kidney disease at various stages in our region. We also aim to investigate the relationship between prolactin levels and N-terminal pro-B-type natriuretic peptide (NT-proBNP), a marker of cardiac function.

## 2. Results

### 2.1. Group Characteristics

Table 1 presents the clinical and laboratory characteristics of the total group of 157 patients. The median age was 66 years; 25% of the population was younger than 57 years, while another 25% were older than 74 years. The majority were male, approximately 29% had diabetes, and over three-quarters had hypertension.

The levels of prolactin, NT-proBNP, and inflammatory markers in the total group and the dialysis and pre-dialysis subgroups of patients are presented in Table 2. Prolactin, tumor necrosis factor-alpha (TNF-α), Interleukin 6 (IL-6), Interleukin-1 beta (IL-1β), and NT-proBNP were statistically significantly higher in dialysis than pre-dialysis patients.

Considering the reference limits for serum prolactin established by the laboratory where the determinations were made (non-pregnant women: 2–23 ng/mL, postmenopausal women: 3–15 ng/mL and men: 2–15 ng/mL), we identified hyperprolactinemia in 7.25% (3 women and 2 men) of patients in pre-dialysis stages and 43.2% (18 women and 18 men) among HD patients.

### 2.2. Correlations and Multivariate Analysis on the Total Group

In Table 3, Spearman correlation coefficients for prolactin with the other continuous characteristics in the total group and the subgroups of patients categorized by CKD stage are presented.

Multivariate analysis is presented in Table 4 for the total group, dialysis patients, and pre-dialysis patients, respectively. Log-transformed prolactin was taken as the dependent variable. Age, lean tissue mass (LTM), diastolic blood pressure (DBP), pulse pressure (PP), triglycerides, high-density lipoproteins (HDL)-cholesterol, fasting glucose, corrected calcium, phosphorus, intact parathyroid hormone (iPTH), hemoglobin, ferritin, white blood cell (WBC), IL-1β, NT-proBNP, TNF-α, IL-6, and eGFR were entered in the multivariate model as independent variables when we applied the model to the total group. The variables that were statistically significant in the multivariate model and that were associated with prolactin are presented in Table 4. Ferritin and IL-6 entered the multivariate model as independent variables when we applied the model to the pre-dialysis group. DBP, triglycerides, and HDL-cholesterol were entered in the multivariate model as independent variables when we applied the model to the dialysis group.

### 2.3. Significant Correlations by Subgroups

#### 2.3.1. Correlations According to Diabetes

Prolactin was statistically significantly higher in the group without diabetes [median (25th–75th percentile): 15.12 (7.07; 31.14)] compared to the group with diabetes [7.24 (4.35; 13.18), *p* = 0.011].

Table 5 presents the correlations between prolactin and other continuous variables, as well as the results of multivariate analyses with log-transformed prolactin as the dependent variable in both diabetic and non-diabetic groups. In the multivariate model, only variables that were statistically significant in the bivariate analysis were entered, and the significant predictors were reported in Table 5.

#### 2.3.2. Correlations According to Sex

Prolactin was higher in the female group [median (25th–75th percentile): 12.72 (5.93; 29.04)] compared with the male group [11.34 (4.61; 20.85)], but the difference was not statistically significant (*p* = 0.162).

Table 6 presents the correlations between prolactin and other continuous variables, as well as the results of multivariate analyses with prolactin as the dependent variable separately in female and male subgroups. In the multivariate model, only variables that were statistically significant in the bivariate analysis were entered, and the significant predictors are reported in Table 6.

#### 2.3.3. Correlations According to Obesity

Prolactin was lower in the obese group [median (25th–75th percentile): 11.09 (6.13; 21.1)] compared with the other patients [12.06 (4.83; 29.65)], but the difference was not statistically significant (*p* = 0.274).

Table 7 presents the correlations between prolactin and other continuous variables, as well as the results of multivariate analyses with log-transformed prolactin as the dependent variable separately in the obese and non-obese subgroups.

#### 2.3.4. Correlations According to Hypertension

Prolactin was statistically significantly lower in the hypertension group [median (25th–75th percentile): 10.51 (4.72; 17.5)] compared to patients without hypertension [21.5 (13.71; 36.23), *p* < 0.001].

Table 8 presents the correlations between prolactin and other continuous variables, as well as the results of multivariate analyses with log-transformed prolactin as the dependent variable, separately in subgroups with and without hypertension.

## 3. Discussion

The main results of our study indicate that inflammatory syndrome biomarker IL-1 beta, muscular mass, and NT proBNP were the most significant factors associated with prolactin levels in chronic kidney disease. We observed that elevated prolactin levels were associated with increased NT-proBNP values, a biomarker of cardiac function. Patients without diabetes and hypertension exhibited significantly higher prolactin levels, but gender and obesity did not considerably impact prolactin values. The increase in prolactin levels was correlated with a decrease in estimated glomerular filtration rate, suggesting that prolactin levels rise as chronic kidney disease progresses. We identified hyperprolactinemia in fewer than 10% of patients in the pre-dialysis stage and almost 50% of hemodialysis patients, consistent with other data for HD patients. However, this was a significantly lower percentage in pre-dialysis CKD compared with another study [15], a particular characteristic of our population study. Prolactin levels in our dialysis patients were considerably higher than in those at pre-dialysis CKD stages.

Regarding the relationship between prolactin and markers of inflammatory syndrome, it is known that this hormone is involved in the immune response and can act as a proinflammatory cytokine [21,22]. Parameters indicative of chronic inflammation, such as high-sensitivity C-reactive protein, interleukin-6, and TNF-α, are significantly and independently associated with increased serum prolactin concentrations in type 2 diabetes, chronic kidney disease, and chronic heart failure [23,24,25]. Similarly, our study found that, in the entire group of patients with CKD, high prolactin values were associated with elevated levels of IL-6, TNF-α, and ferritin, but not with high-sensitivity C-reactive protein, except in diabetic patients. However, the most significant association in our study between prolactin and an inflammatory biomarker was with IL-1β. High prolactin levels were associated with low levels of IL-1β in multivariate analysis. There are multiple explanations for this. First, IL-1β can significantly inhibit thyrotropin-releasing hormone-stimulated prolactin secretion [26]. Second, IL-1β, IL-2, and IL-4 decrease prolactin messenger ribonucleic acid levels [27]. Therefore, the relationship between prolactin secretion and cytokines is quite complex. Lymphocytes, thymocytes, and natural killer cells of the immune system can produce prolactin [28]. Prolactin expression in T lymphocytes is stimulated by cyclic adenosine monophosphate, retinoic acid, and calcitriol [28,29,30]. TNF-α stimulates prolactin production [30,31] and can activate the *human prolactin gene promoter* through nuclear factor-kappa B signaling [31]. These mechanisms explain the positive correlation in our study between TNF-α and prolactin. Together, these processes describe why some cytokines inhibit and others stimulate prolactin secretion.

Inflammatory processes may then favor the cardiovascular effects of prolactin [32]. Thus, the proinflammatory environment appears to trigger the proteolytic cleavage of 23-Da prolactin, under the influence of various metalloproteinases, bone morphogenetic protein-1, and the enzyme cathepsin D, into a cardiotoxic 16-kDa fragment [33,34]. This fragment promotes endothelial damage and impairs cardiomyocyte metabolism and contractility [35]. Additionally, prolactin can stimulate the proliferation of vascular smooth muscle cells [36]. Prolactin receptors are located on macrophages found in atherosclerotic plaques, suggesting that prolactin receptor signaling plays a role in the local inflammatory response, thus contributing to atherogenesis [37]. All these aspects indicate that hyperprolactinemia can directly and indirectly affect the cardiovascular system [38], altering vascular tone [39] and impairing cardiac contractility.

We observed an association between elevated prolactin levels and high NT-proBNP values, a marker of cardiac function, as reported in other studies in patients with chronic kidney disease [40], in the entire group and several subgroups: men, non-obese patients, and patients with and without diabetes or hypertension. This relationship provides data that may contribute to elucidating the pathogenesis of heart failure in CKD. To validate this association, studies examining the relationship between prolactin levels and echocardiographic markers of heart failure, as well as the relationship between prolactin and NT-proBNP, are needed. However, NT-proBNP in CKD is influenced not only by cardiomyocyte function [41] but also by other factors, including hydration status, eGFR, and anemia [42,43]. These factors must be considered when interpreting the relationship described above. NT-proBNP, although influenced by factors other than cardiac function, has demonstrated significant cardiovascular prognostic value in patients with varying degrees of renal dysfunction [44]. The association of prolactin with NT-proBNP suggests that therapy reducing prolactin levels may decrease NT-proBNP and could be beneficial in heart failure patients with hyperprolactinemia and CKD. In other pathological conditions with hyperprolactinemia, such as prolactinomas, the relationship of prolactin with cardiovascular disease has also been observed [45,46], and therapies that reduce prolactin levels may modify cardiovascular pathology in these patients [47].

Additionally, we found a significant correlation between hyperprolactinemia and elevated diastolic blood pressure in our dialysis patients. The link between dopamine and prolactin could explain this association. The inhibitory effect of hypothalamic dopamine mainly regulates prolactin secretion. When dopamine synthesis decreases, prolactin levels increase, which in turn increases sympathetic tone and promotes the development of hypertension [48]. Then, prolactin can modulate endothelial nitric oxide synthase activity and influence blood pressure [49].

Regarding the relationship between prolactin and anthropometric parameters, we observed an association between high prolactin levels and reduced muscle mass in the total group, as well as in several subgroups, including hypertensive patients and non-diabetic patients. Increased prolactin levels secondary to CKD can cause reduced testosterone levels and, through this mechanism, reduced muscle mass [50]. Our study also showed an association between high body mass index (BMI) and high prolactin in non-obese patients, but interestingly, we found no direct correlation between prolactin levels and fat mass, although prolactin is secreted by human adipocytes and is considered an adipokine [51]. The concentration of prolactin released from adipocytes is lower than that from the pituitary gland, and this additional release of prolactin may influence serum levels in individuals with morbid obesity [52]. This mechanism is not dominant in our patients. In pre-dialysis chronic kidney disease (CKD) patients, a direct correlation between prolactin and fat mass was observed in men in another study [53].

It is well established that hyperprolactinemia is associated with insulin resistance and the metabolic syndrome [15]. The relationship between prolactin and metabolism is a complex one. Elevated prolactin levels reduce lipoprotein lipase activity, which is associated with hypogonadism, weight gain, and dyslipidemia [7,54,55]. However, previous studies have yielded mixed results, with some indicating an inhibition of lipogenesis under the influence of prolactin, while others demonstrate promotion of lipogenesis [56,57,58]. Prolactin levels in our patients were associated with HDL cholesterol in dialysis patients and correlated with triglycerides in dialysis patients, too, but not with low-density lipoprotein (LDL) cholesterol. In patients without chronic kidney disease, elevated prolactin levels lead to changes in the lipid profile, increasing LDL-cholesterol and triglycerides while decreasing HDL [59,60]. The metabolic effects of prolactin appear to be particularly pronounced in men, possibly due to its hypogonadotropic effects [61].

Another metabolic effect of prolactin is the stimulation of insulin secretion from pancreatic beta cells, where specific receptors for this hormone are found. Therefore, hyperprolactinemia is associated with increased insulin secretion [62], and consequently, a decrease in glycemic values, as observed in our study in the women subgroup. It has been noted that higher prolactin levels in older individuals without diabetes are associated with improved insulin sensitivity and lower glycemic values. This relationship is reversed in younger people [62]. Both high and low prolactin levels are associated with the risk of diabetes [63]. Restoring normal prolactin levels with appropriate therapy has improved metabolic function [64]. Accordingly, treating hyperprolactinemia in patients without CKD with dopamine agonists results in weight loss, increased insulin sensitivity, and improved lipid profiles [65,66]. The metabolic effect of this drug on CKD secondary hyperprolactinemia is not yet known.

In our CKD patients, prolactin has also been associated with markers of mineral and bone metabolism in univariate analysis, as reported in other studies [67,68]. Prolactin appears to influence both osteoblast and osteoclast activity through different pathways. It has a direct inhibitory effect on osteoblast function [67], upregulates markers of bone formation, such as runt-related transcription factor 2 and alkaline phosphatase, in the early stages of osteoblast differentiation [69], and downregulates osteoprotegerin protein expression in osteoblasts. Prolactin also increases levels of receptor activator NF-kB ligand, a key factor in osteoclast formation, resulting in higher bone turnover rates [70]. It upregulates osteoclastogenic modulators, such as monocyte chemoattractant protein-1, cyclooxygenase-2, TNF-α, IL-1, and nephrin-B1, and may also have a direct effect on osteoclasts [71]. Perhaps through this mechanism, in our study, iPTH—a marker of high bone turnover—and phosphate were directly associated with elevated prolactin levels. Patients with elevated PTH levels have been shown to have higher prolactin levels [72]. At low concentrations, prolactin appears to reduce osteoclast activity [73].

Our study’s age-related variation in prolactin was similar to that reported in some studies of patients without chronic kidney disease [74]. According to some authors, mean prolactin levels were significantly higher in younger individuals (under 30 years of age) compared to older individuals (30 years or older) [65,75,76]. Our study also found an increased prolactin value in young people, especially in young patients without diabetes. This variation may be secondary to age-related changes in the dopaminergic regulation of prolactin secretion, which have also been reported in other studies [77,78].

Prolactin may increase overall mortality [79] and cardiovascular mortality in HD patients [15,80,81,82], possibly through the same type of disorders identified in our study. For some of these, prolactin-lowering therapy has already been tried with favorable results in the non-CKD population [83]. It remains to be seen whether these therapies will be effective in patients with CKD and hyperprolactinemia.

Our study has some limitations. First, because it is cross-sectional and observational, we could only assess associations between various parameters rather than pathogenic mechanisms. Second, the study includes a relatively small number of patients, but the statistical approach is valid. Third, we did not have a control group; however, we made comparisons between subgroups, and we consider the results relevant. Our findings may pave the way for further studies with a larger sample size.

## 4. Materials and Methods

### 4.1. Patients

We conducted a cross-sectional, observational, single-center study on 157 randomly selected patients aged 18–90 years with CKD at various stages, classified according to the Kidney Disease Improving Global Outcomes (KDIGO) guidelines [84]. We included pre-dialysis patients from the Department of Nephrology at the Cluj County Emergency Clinical Hospital and HD patients from the Nefromed Dialysis Center in Cluj-Napoca. Some of the patients, those in the HD group, participated in another study [85]. These patients were described there.

For HD patients, additional inclusion criteria were a minimum of 6 months of maintenance HD and the absence of residual renal function. HD patients received HD three times a week, for 4–5 h per session. We excluded patients who had acute inflammatory processes, terminal malignancies, prior renal transplants, or were receiving immunosuppressive treatment, and patients hospitalized with acute heart failure [85].

All patients included in the study met the inclusion and exclusion criteria and provided informed consent. All procedures adhered to the ethical standards set by the institutional and national research committees, the 1964 Declaration of Helsinki, and its subsequent amendments. The Ethics Committee of “Iuliu Hatieganu” University of Medicine and Pharmacy in Cluj-Napoca approved the study. Demographic data and information on comorbid conditions (such as diabetes, hypertension, cardiovascular disease, hepatitis B or C infection, smoking status, and treatment) were collected from their medical records. Additionally, we recorded clinical data, including age, weight, height, systolic blood pressure (SBP), diastolic blood pressure (DBP) (pre-dialysis values), and a history of cardiovascular disease.

For calculated BMI, we use the formula BMI = weight (kg)/height^2^ (m^2^), and for PP, the formula PP = SBP − DBP (mmHg).

### 4.2. Laboratory Parameters

All biochemical analyses were performed after an overnight fast, between 7:00 and 9:00 a.m., and for HD patients, always on a midweek non-dialysis day. Current measurements at the initiation of this study include serum electrolytes, albumin, creatinine, uric acid, iron profile (iron, transferrin, and ferritin), lipid profile (total cholesterol, triglycerides, and HDL-cholesterol), high-sensitivity C-reactive protein, alkaline phosphatase, and iPTH. The serum was separated using centrifugation at 10,000 rotations per minute for 3 min. Samples for fasting glucose, total cholesterol, high-density lipoprotein (HDL) cholesterol, triglycerides, calcium, and phosphate were analyzed using an automated colorimetric enzymatic method. Parathormone levels were measured with an electrochemiluminescence immunoassay (ECLIA method). A standard hematology panel was conducted using automated flow cytometry, while hs C-reactive protein (CRP) and albumin levels were assessed using an immunoturbidimetric method. All samples were shipped and promptly evaluated by the same authorized laboratory. For the measurement of prolactin, NT-proBNP, IL-6, IL-1β, and TNF-α, a sample of the venous blood was centrifuged, and the serum was refrigerated at −80° Celsius in triplicate Eppendorf tubes until thawed for analysis, which was conducted within two weeks after blood collection. We have determined the levels of serum prolactin, NT-proBNP, IL-6, IL-1β, and TNF-α using enzyme-linked immunosorbent assay (ELISA) with commercially available kits (R&D Systems, Minneapolis, MN, USA). The minimum detection limit for prolactin was 1.5 ng/mL, for TNF-α was 15.6 pg/mL, for IL-6, 3.2 pg/mL, for IL-1β, 10.2 pg/mL, and for NT-proBNP was 7.8 pg/mL. For IL-1 beta, the intra-assay coefficient of variation was 5.1% and the inter-assay coefficient of variation was 8.6%. For IL-6, the intra-assay coefficient of variation was 2.7% and the inter-assay coefficient of variation was 3.6%. For TNF alpha, the intra-assay coefficient of variation was 2.6% and the inter-assay coefficient of variation was 7.7% For NT-proBNP, the intra-assay coefficient of variation was <10% and the inter-assay coefficient of variation was <10%. A minimum of three samples of known concentration were tested on one plate to assess intra-assay and inter-assay precision. For pre-dialysis patients, we calculated eGFR using the CKD Epidemiology Collaboration formula [86]. For HD patients, pre-dialysis and post-dialysis urea levels were used to calculate Kt/V, a parameter that measures dialysis adequacy, where K represents the dialyzer’s urea clearance, t is the total dialysis time, and V is the urea distribution volume. Serum calcium was corrected for albumin according to the formula: Corrected calcium (mg/dL) = serum calcium (mg/dL) + 0.8 × [4.0 − serum albumin (g/dL)].

We registered anthropometric parameters in addition to BMI, assessed by bioimpedance using the Body Composition Monitor, a certified device (manufactured by Fresenius Medical Care, Bad Homburg, Germany) that provided body composition as follows: lean tissue mass (LTM) (kg) and adipose tissue mass (ATM) (kg) [87].

For HD patients, nephrologist dialysis prescriptions were necessary to achieve a value of Kt/V ≥ 1.4. All patients were dialyzed with single-use synthetic (polysulphone) dialyzers and heparin as the standard anticoagulant. Erythropoietin was prescribed via a standardized algorithm. Antihypertensive drugs were prescribed for patients whose post-dialysis or inter-dialysis blood pressure was persistently above 150/90 mmHg at dry weight.

### 4.3. Statistical Method

Data were explored in total groups and subgroups. Quantitative variables following a normal distribution were presented as mean ± standard deviation, whereas non-normally distributed variables were reported as median with interquartile range (25th–75th percentile). To enhance statistical robustness in the presence of extreme values and asymmetry in the distribution of the dependent variable, either parametric or non-parametric methods were applied, or the data were normalized using a logarithmic transformation. Categorical variables were expressed as absolute and relative frequencies. We divided the patients into groups based on dialysis, diabetes, gender, obesity, and hypertension. For each group and subgroup, the correlation between prolactin and the other characteristics was analyzed by calculating the Spearman correlation, as prolactin was not normally distributed. The statistical significance of the Spearman correlation coefficient was also reported. The normal distribution was tested using the Shapiro–Wilk test. Prolactin was compared between subgroups (dialysis versus pre-dialysis, diabetes versus non-diabetes, female versus male, obese versus non-obese, and hypertension versus non-hypertension) using the *t* test for independent samples. Multivariate linear regression models with an intercept (enter method) were performed to identify statistically significant factors associated with prolactin levels (log-transformed) in each group and its subgroups. Only those variables that were significantly related to prolactin were taken into the analysis. Each multivariate model was checked for multicollinearity, and variables with a Variance Inflation Factor higher than 5 were excluded from the model. The coefficient and its 95% confidence interval were reported. We reported the *p* value from the *t* test for the significance of beta coefficients. For multivariate analysis, only variables with complete data (i.e., more than 90% of the data) were included in the study. A *p* less than 0.05 was considered statistically significant. SPSS 25.0 was used for the statistical analysis.

## 5. Conclusions

In conclusion, our study demonstrated that prolactin in patients with chronic kidney disease is a hormone associated with numerous metabolic pathways. It is associated with inflammatory molecules and with glycemic and lipid disorders, as well as markers of bone turnover and muscle mass. Prolactin was also strongly correlated with the NT-proBNP marker of cardiac function. The varying results regarding prolactin associations in subgroups of patients highlight the importance of personalized analysis of prolactin connections for ensuring effective, targeted treatment.

## Figures and Tables

**Table 1:** Clinical characteristics of the total group of patients.

Parameters	Total Group (n = 157)
Age (years)	66 (56.5; 74)
Male, no. (%)	86 (54.8)
Diabetes mellitus, no. (%)	46 (29.3)
Hypertension, no. (%)	123 (78.3)
SBP (mmHg)	144.04 ± 21.65
DBP (mmHg)	80 (70; 89)
PP (mmHg)	61 (50; 75)
Body mass index (kg/m^2^)	28.15 (24.46; 30.93)
LTM (kg)	32.32 (25.67; 42.05)
ATM (kg)	41.17 ± 15.96
Dialysis duration (months)	65 (24; 87)
eGFR (mL/min/1.73 m^2^)	27.5 (15; 42)
Kt/V	1.54 ± 0.32
Triglycerides (mg/dL)	130.5 (94.05; 176)
LDL-cholesterol (mg/dL)	100.73 ± 35.26
Total cholesterol (mg/dL)	174.75 ± 39.2
HDL-cholesterol (mg/dL)	40.66 (32; 48)
Fasting glucose (mg/dL)	97 (89; 121.73)
Corrected Calcium (mg/dL)	9 (8.45; 9.44)
Phosphorus (mg/dL)	4.4 (3.45; 5.5)
Alkaline phosphatase (UI/L)	78 (60; 96)
iPTH (pg/mL)	208.3 (108; 398.9)
Hemoglobin (g/dL)	11.8 (10.8; 13)
Serum albumin (g/L)	3.89 ± 0.37
Ferritin (ng/mL)	383.8 (136; 682.2)
hs-C reactive protein (mg/dL)	0.53 (0.23; 1.25)
White blood cells (n/mm^3^)	6675 (5885; 8435)

n, number; median (25th–75th percentile), arithmetic mean ± standard deviation, no. number. SBP, systolic blood pressure; DBP, diastolic blood pressure; PP, pulse pressure; LTM, lean tissue mass; ATM, adipose tissue mass; eGFR, estimated glomerular filtration rate; LDL, low-density lipoprotein; HDL, high-density lipoproteins; iPTH, intact parathyroid hormone.

**Table 2:** Prolactin, NT-proBNP, and inflammatory markers in the total group and dialysis and pre-dialysis subgroups.

Parameters	Total Group (n = 157)	Dialysis Patients (n = 88)	Pre-Dialysis Patients (n = 69)	*p* *
Prolactin (ng/mL)	12 (5.54; 27.16)	19.46 (12.72; 36.16)	4.83 (3.07; 7.74)	<0.001
TNF-α (pg/mL)	226.9 (4.8; 293.86)	283.24 (241.91; 359.83)	4.4 (2.94; 6.47)	0.001
IL-6 (pg/mL)	196.46 (2.62; 285.51)	272.45 (222.41; 342.71)	2.38 (1.7; 3.27)	<0.001
IL-1β (pg/mL)	18.25 (6.92; 128)	47.15 (16.01; 287.57)	7.06 (6.38; 12.77)	<0.001
NT-proBNP (pg/mL)	6795.8 (384.7; 9611.33)	9557.23 (8684.94; 10,006.81)	322.22 (219.11; 560.56)	<0.001

* *t* test for independent samples for unequal variances was used, median (25th–75th percentile). TNF-α, tumor necrosis factor-alpha; IL-6, Interleukin 6; IL-1β, Interleukin-1 beta; NT-proBNP, N-terminal pro-B-type natriuretic peptide.

**Table 3:** Correlations of prolactin with the other continuous characteristics in the total group and in the subgroups of patients.

Parameters Prolactin	Total Group			Pre-Dialysis		Dialysis	
	SCC	*p*	N	SCC	*p*	SCC	*p*
Age (years)	−0.32	<0.001	156	−0.21	0.079	−0.04	0.687
LTM [kg]	−0.22	0.009	140	−0.02	0.876	−0.07	0.535
DBP (mmHg)	−0.25	0.002	156	0.03	0.818	0.26	0.014
PP(mmHg)	0.16	0.041	156	0.10	0.408	−0.03	0.769
Triglycerides (mg/dL)	−0.03	0.693	134	0.28	0.062	−0.23	0.033
HDL-cholesterol (mg/dL)	0.11	0.202	126	0.22	0.184	0.27	0.010
Fasting glucose (mg/dL)	−0.18	0.035	143	−0.01	0.964	−0.06	0.596
Corrected Calcium (mg/dL)	−0.22	0.006	151	−0.24	0.059	0.05	0.627
Phosphorus (mg/dL)	0.36	<0.001	147	0.24	0.072	0.15	0.175
iPTH (pg/mL)	0.36	<0.001	141	0.21	0.124	0.01	0.897
Hemoglobin (g/dL)	−0.28	<0.001	152	−0.14	0.286	−0.07	0.490
Ferritin (ng/mL)	0.46	<0.001	132	0.30	**0.050**	0.00	0.984
WBC (n/mm^3^)	−0.23	0.004	152	0.19	0.128	−0.19	0.078
IL-1β (pg/mL)	0.25	0.002	157	−0.06	0.619	−0.20	0.060
NT-proBNP	0.61	<0.001	157	0.11	0.357	−0.01	0.941
TNF-α(pg/mL)	0.65	<0.001	157	0.21	0.078	0.15	0.168
IL-6 (pg/mL)	0.66	<0.001	157	0.26	**0.033**	0.16	0.130
eGFR (mL/min/1.73 m^2^)	−0.60	<0.001	66	−0.60			

SCC, Spearman correlation coefficient; DBP, diastolic blood pressure; PP, pulse pressure; LTM, lean tissue mass; eGFR, estimated glomerular filtration rate; iPTH, intact parathyroid hormone; TNF-α, tumor necrosis factor-alpha; IL-6, Interleukin 6; IL-1β, Interleukin-1 beta; NT-proBNP, N-terminal pro-B-type natriuretic peptide; WBC, white blood cell.

**Table 4:** Multivariate linear regressions with log-transformed prolactin as the dependent variable and significant variables in bivariate analysis in the total group and the subgroups of patients.

Parameters Prolactin	Total Group Patients		Pre-Dialysis Patients		Dialysis Patients	
	B, 95%CI *	*p*	B, 95%CI *	*p*	B, 95%CI *	*p*
LTM [kg]	−0.03 (−0.04; −0.01)	0.003				
DBP (mmHg)					0.014 (0.001; 0.027)	0.029
HDL-cholesterol (mg/dL)					0.012 (0.001; 0.024)	0.038
IL-1β (pg/mL)	−0.001 (−0.001; −0.00003)	0.037				
NT-proBNP (pg/mL)	0.0001 (0.0001; 0.0001)	<0.001				

* For multivariate analysis, only variables with complete data (more than 90% of the data) and statistically significant were taken in the analysis as independent variables; prolactin was log-transformed. CI, confidence interval; LTM, lean tissue mass; IL-1β, Interleukin-1 beta; NT-proBNP, N-terminal pro-B-type natriuretic peptide.

**Table 5:** Correlations and multivariate linear regression models of prolactin/log-transformed prolactin with the other continuous characteristics in subgroups of patients with and without diabetes.

Prolactin	With Diabetes (n = 46)				Without Diabetes (n = 108)			
	Bivariate		Multivariate *		Bivariate		Multivariate *	
	SCC	*p*	B, 95%CI	*p*	SCC	*p*	B, 95%CI	*p*
Age (years)	−0.27	0.076			−0.28	0.003	−0.019 (−0.035; −0.003)	0.021
DBP (mmHg)	−0.32	0.031			−0.13	0.176		
PP (mmHg)	0.42	0.005			0.18	0.058		
LTM (kg)	−0.23	0.149			−0.26	0.011	−0.026 (−0.048; −0.004)	0.022
eGFR (mL/min/1.73 m^2^)	−0.49	0.006			−0.74	<0.001		
Corrected Calcium (mg/dL)	−0.25	0.111			−0.25	0.011		
Phosphorus (mg/dL)	0.33	0.034			0.34	<0.001		
iPTH (pg/mL)	0.40	0.011			0.33	0.001		
Hemoglobin (g/dL)	−0.09	0.540			−0.43	<0.001		
hs-C reactive protein (mg/dL)	0.43	0.004			0.03	0.759		
Ferritin (ng/mL)	0.59	<0.001			0.41	<0.001		
WBC (no./mm^3^)	0.06	0.711			−0.22	0.027		
IL-1β (pg/mL)	0.28	0.059			0.26	0.007		
TNF-α (pg/mL)	0.65	<0.001			0.61	<0.001		
IL-6 (pg/mL)	0.69	<0.001			0.59	<0.001		
NT-proBNP (pg/mL)	0.68	<0.001	0.0001 (0.00003; 0.0003)	0.015	0.52	<0.001	0.0001 (0.00001; 0.0001)	0.028

* For multivariate analysis, only variables with complete data (more than 90% of the data) were taken in the analysis as independent variables; prolactin was log-transformed. SCC, Spearman correlation coefficient; CI, confidence interval; DBP, diastolic blood pressure; WBC, white blood cell; PP, pulse pressure; LTM, lean tissue mass; eGFR, estimated glomerular filtration rate; iPTH, intact parathyroid hormone; TNF-α, tumor necrosis factor-alpha; IL-6, Interleukin-6; IL-1β, Interleukin-1 beta; NT-proBNP, N-terminal pro-B-type natriuretic peptide.

**Table 6:** Correlations and multivariate linear regression models of prolactin/log-transformed prolactin with the other continuous characteristics in subgroups of male and female patients.

Prolactin	Women (n = 71)				Men (n = 86)			
	Bivariate		Multivariate *		Bivariate		Multivariate *	
	SCC	*p*	B, 95%CI	*p*	SCC	*p*	B, 95%CI	*p*
Age (years)	−0.29	0.014			−0.38	<0.001		
Fasting glucose (mg/dL)	−0.40	0.001	−0.01 (−0.02; −0.001)	0.040	−0.04	0.731		
DBP (mmHg)	−0.14	0.233			−0.31	0.004		
Corrected calcium (mg/dL)	−0.40	0.001			−0.13	0.248		
Phosphorus (mg/dL)	0.29	0.017			0.43	<0.001		
iPTH (pg/mL)	0.28	0.026			0.42	<0.001		
Hemoglobin (g/dL)	−0.44	<0.001			−0.15	0.184		
Ferritin (ng/mL)	0.41	0.002			0.47	<0.001		
WBC (no./mm^3^)	−0.24	0.049			−0.23	0.040		
IL-1β (pg/mL)	0.21	0.076			0.29	0.006		
NT-proBNP (pg/mL)	0.60	<0.001			0.61	<0.001	0.0001 (0.00003; 0.0002)	0.006
TNF-α (pg/mL)	0.60	<0.001			0.73	<0.001		
IL-6 (pg/mL)	0.62	<0.001	0.002 (0.0001; 0.003)	0.042	0.71	<0.001		
eGFR (mL/min/1.73 m^2^)	−0.73	<0.001			−0.46	0.006		

* For multivariate analysis, only variables with complete data (more than 90% of the data) were taken in the analysis as independent variables; prolactin was log-transformed. SCC, Spearman correlation coefficient; CI, confidence interval; WBC, white blood cell; DBP, diastolic blood pressure; eGFR, estimated glomerular filtration rate; iPTH, intact parathyroid hormone; TNF-α, tumor necrosis factor-alpha; IL-6, Interleukin-6 6; IL-1β, Interleukin-1 beta; NT-proBNP, N-terminal pro-B-type natriuretic peptide.

**Table 7:** Correlations and multivariate linear regression models of prolactin/log-transformed prolactin with the other continuous characteristics in subgroups of patients with and without obesity.

Prolactin	BMI ≥ 30 (n = 51)				BMI < 30 (n = 103)			
	Bivariate		Multivariate *		Bivariate		Multivariate *	
	SCC	*p*	B, 95%CI	*p*	SCC	*p*	B, 95%CI	*p*
Age (years)	−0.15	0.283			−0.35	<0.001		
Body mass index (kg/m^2^)	0.21	0.130			−0.26	0.007		
LTM (kg)	−0.36	0.014			−0.17	0.111		
DBP (mmHg)	−0.42	0.002			−0.20	0.044		
PP (mmHg)	0.32	0.022			0.10	0.299		
HDL-cholesterol (mg/dL)	−0.40	0.009			0.33	0.003	0.02 (0.006; 0.04)	0.008
Fasting glucose (mg/dL)	−0.33	0.021			−0.07	0.486		
Corrected calcium (mg/dL)	−0.22	0.121			−0.22	0.031		
Phosphorus (mg/dL)	0.46	0.001			0.28	0.006		
iPTH (pg/mL)	0.60	<0.001			0.24	0.023		
Hemoglobin (g/dL)	−0.31	0.028			−0.25	0.011		
Ferritin (ng/mL)	0.43	0.004			0.51	<0.001		
WBC (no./mm^3^)	−0.27	0.057			−0.23	0.022		
IL-1β (pg/mL)	0.27	0.057			0.23	0.017		
NT-proBNP (pg/mL)	0.71	<0.001			0.56	<0.001	0.0001 (0.00003; 0.0002)	0.003
TNF-α (pg/mL)	0.78	<0.001			0.59	<0.001		
IL-6 (pg/mL)	0.72	<0.001			0.62	<0.001		
eGFR (mL/min/1.73 m^2^)	−0.54	0.010			−0.63	<0.001		

* For multivariate analysis, only variables with complete data (more than 90% of the data) were taken in the analysis as independent variables; prolactin was log-transformed. SCC, Spearman correlation coefficient; CI, confidence interval; WBC, white blood cell; DBP, diastolic blood pressure; PP, pulse pressure; LTM, lean tissue mass; eGFR, estimated glomerular filtration rate; HDL, high-density lipoproteins; iPTH, intact parathyroid hormone; TNF-α, tumor necrosis factor-alpha; IL-6, Interleukin-6 6; IL-1β, Interleukin-1 beta; NT-proBNP, N-terminal pro-B-type natriuretic peptide.

**Table 8:** Correlations and multivariate linear regression models of prolactin/log-transformed prolactin with the other continuous characteristics in subgroups of patients with and without hypertension.

Prolactin	With Hypertension (n = 123)				Without Hypertension (n = 33)			
	Bivariate		Multivariate *		Bivariate		Multivariate *	
	SCC	*p*	B, 95%CI	*p*	SCC	*p*	B, 95%CI	*p*
Age (years)	−0.32	<0.001			−0.34	0.053		
LTM (kg)	−0.21	0.028			−0.11	0.550		
DBP (mmHg)	−0.32	<0.001			0.27	0.129		
Corrected calcium (mg/dL)	−0.24	0.008			0.05	0.788		
Phosphorus (mg/dL)	0.42	<0.001			0.20	0.294		
iPTH (pg/mL)	0.37	<0.001			0.11	0.594		
Fasting glucose (mg/dL)	−0.04	0.689			−0.47	0.007		
Ferritin (ng/mL)	0.55	<0.001			0.36	0.062		
WBC (no./mm^3^)	−0.18	0.047			0.00	0.979		
Hemoglobin (g/dL)	−0.26	0.004			−0.27	0.136		
Bicarbonate level (mEq/L)	0.24	0.033			−0.18	0.364		
IL-1β (pg/mL)	0.30	0.001			0.01	0.971		
NT-proBNP (pg/mL)	0.61	<0.001	0.0001 (0.00005; 0.0002)	**0.001**	0.56	0.001	0.0001 (0.00003; 0.0002)	0.012
TNF-α (pg/mL)	0.70	<0.001			0.33	0.062		
IL-6 (pg/mL)	0.68	<0.001			0.46	0.007		
eGFR (mL/min/1.73 m^2^)	−0.53	<0.001			−0.94	0.005		

* For multivariate analysis, only variables with complete data (more than 90% of the data) were taken in the analysis as independent variables; prolactin was log-transformed. SCC, Spearman correlation coefficient; CI, confidence interval; WBC, white blood cell; DBP, diastolic blood pressure; LTM, lean tissue mass; eGFR, estimated glomerular filtration rate; iPTH, intact parathyroid hormone; TNF-α, tumor necrosis factor-alpha; IL-6, Interleukin-6; IL-1β, Interleukin-1 beta; NT-proBNP, N-terminal pro-B-type natriuretic peptide.

## Data Availability

The research data that support the findings of this study are not publicly available. Further inquiries can be directed to the corresponding author.

## Abbreviations

The following abbreviations are used in this manuscript:

CKD	Chronic kidney disease
HD	Hemodialysis
SBP	Systolic blood pressure
DBP	Diastolic blood pressure
PP	Pulse pressure
BMI	Body mass index
LTM	Lean tissue mass
ATM	Adipose tissue mass
eGFR	Estimated glomerular filtration rate
LDL	Low-density lipo-protein
HDL	High-density lipoproteins
iPTH	Intact parathyroid hormone
TNF-α	Tumor necrosis factor-alpha
IL-6	Interleukin 6
IL-1β	Interleukin-1 beta
NT-proBNP	N-terminal pro-B-type natriuretic peptide
SCC	Spearman correlation coefficient
WBC	White blood cell
CI	Confidence interval
